# Neonatal cardiopulmonary resuscitation: is ROSC enough?

**DOI:** 10.1038/s41390-024-03398-8

**Published:** 2024-07-24

**Authors:** Robert M. Dietz, Fernando F. Gonzalez

**Affiliations:** 1 Department of Pediatrics, Children’s Hospital of Colorado, University of Colorado Anschutz Medical Campus, Aurora, CO USA; 2 Department of Pediatrics, UCSF Benioff Children’s Hospital, University of California San Francisco, San Francisco, CA USA

The study by Morin et al. examines a critical question with a profound short and long-term outcome after neonatal code events—what is the most effective way to provide cardiopulmonary resuscitation in this population at risk for short and long-term morbidity and mortality? They studied twenty infantile piglets and randomized to continuous chest compressions superimposed with sustained inflation (CC + SI) versus chest compressions with asynchronized ventilation (CCaV), hypothesizing the addition of SI would reduce time to return of spontaneous circulation (ROSC) following asphyxia-induced bradycardic arrest. CC + SI increased intrathoracic pressure, minute ventilation, and reduced duration of resuscitation compared CCaV in this model. The use of a gyrencephalic model of brain development is a strength of this study; however, numbers are small, and the study does not address long-term outcomes or brain injury post-arrest.

Clinical neonatal and pediatric studies show variable responses to different CPR techniques, but a consistent finding is that survival to hospital discharge is often poor. For example, for pediatric in-hospital arrest, as many as 80-90% survive the event but do not survive to hospital discharge, and this is often secondary to neurological status after the code event.^1^ High quality chest compressions, timely defibrillation, and appropriate ventilation, medication, and treatment of underlying cause improve early survival. But importantly, structured post-event care focused on targeted temperature management, hemodynamic optimization, and ICU management are necessary for improved neurologic outcome and survival.^2^ Newborns are distinct from older children in that the cause is most often secondary to respiratory failure leading to hypoxia and bradycardia.^3^ In this setting airway management and ventilation are more beneficial, but optimal CPR strategies and impact on long-term outcome are not clear.^4–6^

A number of preclinical studies have endeavored to answer the question regarding optimal CPR strategy, but importantly, many of these do not directly address post-event care or long-term effects on neurodevelopmental outcomes. Previous piglet studies examining different ventilation strategies with CC found no difference between 3:1 and 15:2 ratios on ROSC or neurologic outcome.^7–9^ In lambs SI was found to increase intrathoracic pressure without impeding blood flow, and optimal CC rate may depend on size and weigh but more CC may be necessary for neonates.^10,11^ While rodents, rabbits, piglets, lambs, primates and other species have been used for newborn animal models, they need to be assessed with the same rigor as human trials.^6^

While neonatal resuscitation strategy remains an important area of study, understanding long-term neurological injury and outcome is essential to improved translational study. Studies relying simply on neuro-histopathology have yet to yield significant therapeutic strategies in children. Neonatal lambs experiencing umbilical cord occlusion and asystole showed severe motor deficits associated with gliosis, inflammation and neuronal death in 35% of their cohort in the six days following resuscitation.^12^ Similarly, a recent porcine model that randomized arrest to 30 or 60 min prior to ECMO cannulation showed that longer durations of CPR were associated with more severe neurologic injury, assessed via various biomarkers and histopathological evaluations following 24 h of ECMO.^13^ Perhaps the best studied preclinical model for long-term outcomes following cardiac arrest is the juvenile mouse. Synaptic plasticity, memory behavior and molecular signaling have been studied up to 30 days after CPR showing significant cognitive impairment in males and females.^14–16^ While rodent models are important to investigate mechanisms of neuronal injury and physiologic deficits following cardiac arrest and resuscitation, large animal models with long-term follow up must be used as part of the preclinical assessment for potential therapeutic strategies.

To address limitations of rodent models in translating to human clinical outcomes, we must prioritize diverse preclinical models that reflect key aspects of neonatal brain injury, such as chronicity and inflammation. Utilizing various animal models, including those with more complex brain structures like ferrets, piglets, and sheep, will provide a more accurate representation of human conditions. The study by Morin et al. (this study) is a great example focusing on resuscitation efforts, albeit without a focus on neurodevelopmental outcomes. Neonatal ferrets have been used recently, using a protocol involving injection of lipopolysaccharide followed by hypoxia at postnatal day 10 .^17^ These kits can then be examined for several weeks following injury to assess behavior, brain MRI, network connectivity and immunohistochemistry. Likewise, newborn piglet asphyxia models have been used over the past 15 years to study the effect of melatonin,^18^ dexmedetomidine,^19^ anesthetics,^20,21^ and concomitant inflammation^22^ on brain injury. While these piglet experiments concluded 48 h following resuscitation and provided critical information on neuro-histopathologic injury, neurodevelopmental outcomes were rarely studied. A recent study that highlights how survival following resuscitation in a newborn lamb examined the therapeutic potential of azithromycin administered peri- and postnatally.^23^ Not only did administration of azithromycin result in earlier return of spontaneous circulation, improved inflammatory profiles, and higher neurobehavior scores at seven days of life, but important pharmacokinetic information was gained in this survival study. Therefore, diverse preclinical models have been developed but it must be underscored that surviving large animals to assess long-term effects requires vast resources that are difficult to achieve under current funding models.

The study by Morin et al. continues an important line of research examining the most effective way to resuscitate newborns. Future studies examining resuscitation techniques should be powered to examine sex-specific differences and use standardized post-ROSC treatment protocols and multimodal assessment to study neurodevelopmental outcomes.^24,25^ As such, several labs are starting to use piglets and lambs designed to study this question, without the variable of different resuscitation models. We advocate for the careful selection of animal models that accurately reflect the pathophysiological nuances of early asystole and outcomes. This approach is essential for refining our protective strategies before progressing to extensive controlled trials.

## References

[1] Morgan, R. W., Kirschen, M. P., Kilbaugh, T. J., Sutton, R. M. & Topjian, A. A. Pediatric in-hospital cardiac arrest and cardiopulmonary resuscitation in the United States: A review. *JAMA Pediatr.* **175**, 293–302 (2021). 33226408 10.1001/jamapediatrics.2020.5039 PMC8787313

[2] Topjian, A. A. et al. Pediatric post-cardiac arrest care: A scientific statement from the American Heart Association. *Circulation* **140**, e194–e233 (2019). 31242751 10.1161/CIR.0000000000000697

[3] Boldingh A. M. et al. Outcomes following neonatal cardiopulmonary resuscitation. *Tidsskr Nor Laegeforen.* 2018;138. 10.4045/tidsskr.17.0358 29808658

[4] Okereafor, A. et al. Patterns of brain injury in neonates exposed to perinatal sentinel events. *Pediatrics* **121**, 906–914 (2008). 18450893 10.1542/peds.2007-0770

[5] Sarnat, H. B. et al. Neonatal encephalopathy following fetal distress. A clinical and electroencephalographic study. *Arch. Neurol.* **33**, 696–705 (1976). 987769 10.1001/archneur.1976.00500100030012

[6] Skåre, C. et al. Incidence of newborn stabilization and resuscitation measures and guideline compliance during the first minutes of life in Norway. *Neonatology* **108**, 100–107 (2015). 26089106 10.1159/000431075

[7] Poulton, D. A. et al. Assessment of chest rise during mask ventilation of preterm infants in the delivery room. *Resuscitation* **82**, 175–179 (2011). 21074926 10.1016/j.resuscitation.2010.10.012

[8] Perlman, J. M. et al. Cardiopulmonary resuscitation in the delivery room. *Assoc. Clin. Events Arch. Pediatr. Adolesc. Med*. **149**, 20–25 (1995). 10.1001/archpedi.1995.02170130022005 7827654

[9] Ikonen, R. S. et al. Hyperbilirubinemia, hypocarbia and periventricular leukomalacia in preterm infants: relationship to cerebral palsy. *Acta Paediatr.* **81**, 802–807 (1992). 1421887 10.1111/j.1651-2227.1992.tb12107.x

[10] Vali, P. et al. Continuous chest compressions during sustained inflations in a perinatal asphyxial cardiac arrest lamb model. *Pediatr. Crit. Care Med.* **18**, e370–e377 (2017). 28661972 10.1097/PCC.0000000000001248 PMC5552419

[11] Schmölzer, G. M. et al. Single versus continuous sustained inflations during chest compressions and physiological-based cord clamping in asystolic lambs. *Arch. Dis. Child Fetal Neonatal Ed.* **107**, 488–494 (2022). 34844983 10.1136/archdischild-2021-322881 PMC9411918

[12] Mike, J. K. et al. Defining longer-term outcomes in an ovine model of moderate perinatal hypoxia-ischemia. *Dev. Neurosci.* **44**, 277–294 (2022). 35588703 10.1159/000525150 PMC9474697

[13] Slovis, J. C. et al. Pediatric extracorporeal cardiopulmonary resuscitation: development of a porcine model and the influence of cardiopulmonary resuscitation duration on brain injury. *J. Am. Heart Assoc.* **12**, e026479 (2023). 36789866 10.1161/JAHA.122.026479 PMC10111482

[14] Dietz, R. M. et al. Juvenile cerebral ischemia reveals age-dependent BDNF-TrkB signaling changes: Novel mechanism of recovery and therapeutic intervention. *J. Cereb. Blood Flow. Metab.* **38**, 2223–2235 (2018). 29611441 10.1177/0271678X18766421 PMC6282214

[15] Dietz, R. M. et al. Therapeutic hypothermia protects against ischemia-induced impairment of synaptic plasticity following juvenile cardiac arrest in sex-dependent manner. *Neuroscience* **325**, 132–141 (2016). 27033251 10.1016/j.neuroscience.2016.03.052 PMC5217704

[16] Dietz, R. M. et al. Functional restoration following global cerebral ischemia in juvenile mice following inhibition of transient receptor potential M2 (TRPM2) ion channels. *Neural Plast.* **2021**, 8774663 (2021). 34659399 10.1155/2021/8774663 PMC8514917

[17] Wood, T. et al. A ferret model of encephalopathy of prematurity. *Dev. Neurosci.* **40**, 475–489 (2018). 31079096 10.1159/000498968 PMC6658350

[18] Robertson, N. J. et al. Melatonin augments hypothermic neuroprotection in a perinatal asphyxia model. *Brain* **136**, 90–105 (2013). 23183236 10.1093/brain/aws285

[19] Ezzati, M. et al. Dexmedetomidine combined with therapeutic hypothermia is associated with cardiovascular instability and neurotoxicity in a piglet model of perinatal asphyxia. *Dev. Neurosci.* **39**, 156–170 (2017). 28391258 10.1159/000458438

[20] Broad, K. D. et al. Surgery increases cell death and induces changes in gene expression compared with anesthesia alone in the developing piglet brain. *PLoS ONE* **12**, e0173413 (2017). 28355229 10.1371/journal.pone.0173413 PMC5371291

[21] Broad, K. D. et al. Isoflurane exposure induces cell death, microglial activation and modifies the expression of genes supporting neurodevelopment and cognitive function in the male newborn piglet brain. *PLoS ONE* **11**, e0166784 (2016). 27898690 10.1371/journal.pone.0166784 PMC5127656

[22] Martinello, K. A. et al. Acute LPS sensitization and continuous infusion exacerbates hypoxic brain injury in a piglet model of neonatal encephalopathy. *Sci. Rep.* **9**, 10184 (2019). 31308390 10.1038/s41598-019-46488-y PMC6629658

[23] Mike, J. K. et al. Perinatal azithromycin provides limited neuroprotection in an ovine model of neonatal hypoxic-ischemic encephalopathy. *Stroke* **54**, 2864–2874 (2023). 37846563 10.1161/STROKEAHA.123.043040 PMC10589434

[24] Ijuin, S. et al. Current animal models of extracorporeal cardiopulmonary resuscitation: A scoping review. *Resuscitation .* **15**, 100426 (2023). 10.1016/j.resplu.2023.100426 PMC10372365 37519410

[25] Yu, S. et al. Rat model of asphyxia-induced cardiac arrest and resuscitation. *Front Neurosci.* **16**, 1087725 (2022). 36685224 10.3389/fnins.2022.1087725 PMC9846144

